# Immunobiotic and Paraprobiotic Potential Effect of *Lactobacillus casei* in a Systemic Toxoplasmosis Murine Model

**DOI:** 10.3390/microorganisms8010113

**Published:** 2020-01-14

**Authors:** Angel Gustavo Salas-Lais, Atzín Robles-Contreras, José Abraham Balderas-López, Victor Manuel Bautista-de Lucio

**Affiliations:** 1 Departamento de Microbiología y Proteómica Ocular de la Unidad de Investigación del Instituto de Oftalmología “Fundación de Asistencia Privada Conde de Valenciana I.A.P.”, Chimalpopoca 14, Obrera, Ciudad de Mexico 06800, Mexico; salas_lais@yahoo.com.mx; 2 Laboratorio de Técnicas Fototérmicas, Departamento de Ciencias Básicas, Unidad Profesional Interdisciplinaria de Biotecnología del Instituto Politécnico Nacional, Acueducto 550, La Laguna Ticomán, Ciudad de Mexico 07340, Mexico; abrahambalderas@hotmail.com; 3 Programa de Posgrado en Biomedicina y Biotecnología Molecular, Escuela Nacional de Ciencias Biológicas, Instituto Politécnico Nacional, Mexico City 11340, Mexico; 4 Área de Imunoproteómica Ocular, Departamento Centro de Investigación Biomédica, Fundación Hospital “Nuestra Señora de la Luz”, Ezequiel Montes 135, Tabacalera, Ciudad de Mexico 06030, Mexico; atzinrc@gmail.com

**Keywords:** *Lactobacillus casei*, immunobiotic, paraprobiotic, activated macrophages, systemic toxoplasmosis, tachyzoites

## Abstract

One of the main characteristics of probiotics is their ability to stimulate and modulate the immune response regardless of their viability. *Lactobacillus casei* (Lc) can stimulate local and systemic immunity, in addition to the activation of macrophages at sites distant from the intestine. Activated macrophages limit the replication of intracellular protozoa, such as *Toxoplasma gondii*, through the production of nitric oxide. The present study aimed to evaluate the protection generated by treatment with viable and non-viable Lc in the murine systemic toxoplasmosis model. CD1 male mice were treated with viable Lc (immunobiotic) and non-viable Lc (paraprobiotic), infected with tachyzoites of *Toxoplasma gondii* RH strain. The reduction of the parasitic load, activation of peritoneal macrophages, inflammatory cytokines, and cell populations was evaluated at 7 days post-infection, in addition to the survival. The immunobiotic and paraprobiotic reduced the parasitic load, but only the immunobiotic increased the activation of peritoneal macrophages, and the production of interferon-gamma (IFN-γ), tumor necrosis factor (TNF), and interleukin-6 (IL-6) while the paraprobiotic increased the production of monocyte chemoattractant protein-1 (MCP-1) and T CD4^+^CD44^+^ lymphocytes. Viable and non-viable Lc increases survival but does not prevent the death of animals. The results provide evidence about the remote immunological stimulation of viable and non-viable Lc in an in vivo parasitic model.

## 1. Introduction

Probiotics are defined by Food and Agriculture Organization and World Health Organization as ‘live microorganisms that when administered in adequate amounts confer benefits to the health of the host’ [1]. An important characteristic of probiotics is the ability to stimulate the local (intestinal mucosa) and distant (systemic) immune response; owing to this, they have been attributed with the ‘immunobiotic term’ [2]. However, there is evidence that the immune stimulation of a probiotic microorganism is independent of the cell viability. In this sense, the term paraprobiotic (ghost probiotics) was proposed to all non-viable microbial cells that, when administered in adequate amounts, confer benefits to the host [3].

Multiple species of the genus *Lactobacillus* have been shown to have the potential to stimulate the innate and adaptive immune response in the host via binding to pattern recognition receptors (PRRs) expressed on immune cells that recognize molecular patterns associated with microorganisms (MAMPs) that triggers the production of cytokines and chemokines [4].

The beneficial effects of *Lactobacillus casei* Shirota (LcS) have been extensively evaluated in clinical trials, demonstrating protection in infectious (diarrhea) and non-infectious (tumor) pathologies. In addition, the beneficial effects of LcS on allergies, irritable bowel disease (IBD), and autoimmune diseases have been confirmed through experimental models. It suggests that LcS not only increases the host’s immune response against cancer and infections but also controls the excessive immune response to inhibit inflammatory processes [5].

Most of the immunostimulation/immunoregulation functions of LcS are involved in immune response cells, such as macrophages and dendritic cells, that carry out antigen recognition and presentation [6]. In addition, macrophages play an important role in the elimination of intracellular pathogenic microorganisms by activating lithic oxygen and nitrogen mechanisms [7]. It has been shown in the murine model that *Lactobacillus casei* ATCC 7469 stimulates the production of nitric oxide (NO), and is capable of eliminating intracellular protozoan parasites, such as *Trypanosoma cruzi* [8], *Plasmodium chabaudi* [9], and *Babesia microti* [10].

One of the most complex intracellular protozoa from an immunological point of view is *Toxoplasma gondii* (*T. gondii*). This protozoan is the causative agent of toxoplasmosis and it is estimated that around one third of the world’s population are infected, whereas pregnant women and immunosuppressed people are more vulnerable to infection [11]. The spread of the parasite through the use of macrophages and dendritic cells known as a ‘Trojan horse’ and the ability to cross the blood–brain and blood–retinal barrier can cause pathologies known as brain toxoplasmosis and chorioretinitis [12].

The pharmacological treatments used in toxoplasmosis, such as clindamycin, pyrimethamine, sulfadiazine, trimetroprim, and sulfamethoxazole, are specific for the control of the tachyzoite stage. However, these drugs can cause side effects and can deteriorate the health of the host [13]. From this perspective, it is necessary to implement new treatments that offer the patient an ability to control the replication of the parasite, increase the immune response, and ensure the integrity of health, such as biotherapies with probiotics.

The work on the murine model carried out by Martínez-Gómez [14] and Ribeiro [15] showed protection in the chronic phase of infection with the low virulence strain ME49 of *T. gondii* when administered with *L. casei* ATCC 7469 and *Bifidobacterium animalis* subspp. *lactis*, respectively, reducing the parasitic load of tissue cysts in the brain. However, there is no evidence that assesses the ability to generate protection by probiotics in high-virulence strains of *T. gondii* infection. Also, *in situ* stimulation of nitric oxide production in macrophages by immunobiotics/paraprobiotics has not been demonstrated.

The objective of the current work was to evaluate the possible immunological protection mechanism of *Lactobacillus casei* as an immunobiotic and paraprobiotic against systemic infection caused by *T. gondii* RH strain in a murine model.

## 2. Materials and Methods

### 2.1. Bacterial Culture and Preparation of Heat Killed Bacteria

The probiotic bacterium *Lactobacillus casei* (Lc) was obtained from the commercial drink Yakult (Yakult^®^, Mexico). *L. casei* was identified by 16S rRNA sequencing. The bacterium was grown in MRS broth (de Man-Rogosa-Sharpe broth, BDL, Franklin Lakes, NJ, USA) at 37 °C at 5% CO_2_ for 24 h. Briefly, the bacteria were harvested and washed twice with sterile phosphate buffered saline (PBS) at 2700× *g* for 15 min and the viable count was made on MRS agar plates at 37 °C with 5% CO_2_ for 24 to 48 h. Colony forming units (CFUs) were quantified and adjusted to 1 × 10^9^ CFU/mL in PBS at pH 7.2; these bacteria were called immunobiotics (IBs). From the same vials adjusted to 1 × 10^9^ CFU/mL, they were subjected to heat treatment at 100 °C for 30 min, immediately after they were placed in an ice bath (thermal shock) [16]. The inactivation was confirmed by the absence of colonies in MRS agar plates; the heat-inactivated bacteria were called paraprobiotics (PPs). 

### 2.2. T. gondii RH

The RH strain of *T. gondii* was maintained in male CD1 mice (12 weeks old) inoculated intraperitoneally with tachyzoites [17]. At 3 days post-infection, a peritoneal wash with sterile PBS was performed and the suspension obtained was centrifuged at 1000× *g* for 10 min. The cell package was resuspended in sterile PBS and the tachyzoites were counted in a Neubauer chamber.

### 2.3. Animal Model

Ethical Committee of Instituto de Oftalmología “Fundación de Asistencia Privada Conde de Valenciana” (CONBIOETICA-09-CEI-023-20160830), approved the use of animals in this study. All animals were treated according to the ARVO statement for the Use of Animals in Ophthalmology and Vision Research. Male CD1 mice aged 8 to 10 weeks and weighing between 28 and 31 g were used in this study and were obtained from the vivarium of the National School of Biological Sciences, National Polytechnic Institute (Mexico City, Mexico). All animals were free of specific parasites and were kept in normal conditions of room temperature, 12/12 h light cycle, and subjected to *ad libitum* fed.

### 2.4. Experimental Design

A batch of 68 male CD1 mice were randomly divided into 4 groups. Three groups were infected with *T. gondii* RH and received a treatment with PBS (*n* = 22), immunobiotics (*n* = 20), or paraprobiotics (*n* = 20). Additionally, a group of uninfected and untreated animals were used as a control group (*n* = 4). The treatment of the animals began on day 0 and concluded on day 14 of the experiment by administering daily intragastrically 100 μL of PBS, 1 × 10^9^ CFU/100 μL of alive Lc (IBs), or 1 × 10^9^ UFC/100 μL of dead Lc (PPs). On day 7, the mice were infected intraperitoneally with 1000 tachyzoites of the *T. gondii* RH strain. Seven to 9 mice per group were sacrificed in a lethal chamber on day 14 of starting treatment, that is, on the 7th day post-infection. The rest of the animals (12 to 13 mice per group) were monitored for survival once the treatment was complete.

### 2.5. Evaluation of Load Tachyzoites T. gondii RH

A first peritoneal wash was performed to obtain ascites fluid with 2 mL of cold sterile PBS, and 1 mL was extracted. It was deposited in a 1.5-mL Eppendorf tube and placed in refrigeration. Then, a second peritoneal wash was performed with 3 mL of cold sterile PBS, and approximately 3 mL of ascites fluid was removed and placed in a Vacutainer tube with heparin (Franklin Lake, NJ, USA). Eppendorf tubes were centrifuged at 3000× *g* for 10 min, and then the cell-free supernatant was removed and stored at −70 °C for the evaluation of inflammatory cytokines. The cell packet was collected and transferred to the tube containing 3 mL of ascites fluid, centrifuged at 3000× *g* for 10 min and the supernatant discarded, and the cell packet was resuspended in 1 mL of sterile PBS. A 1:10 dilution was performed in PBS and the peritoneal exudate cells (PECs) and tachyzoites were quantified in a Neubauer chamber.

### 2.6. In Vivo Cytokine Production

Cytokine concentrations were determined in ascites fluid from CD1 mice. Briefly, the mice were euthanized in a CO_2_ chamber, 2 mL of sterile PBS was inoculated, and a peritoneal wash was performed. The fluid was centrifuged at 10,000× *g* for 10 min at 4 °C. The supernatant was frozen at −70 °C until use. Liquid concentrations of interferon-gamma (IFN-γ), tumor necrosis factor (TNF), monocyte chemoattractant protein-1 (MCP-1), interleukin-6 (IL-6), interleukin-10 (IL-10), and interleukin-12 (IL-12) were quantified with the BD Cytometric Bead Array (CBA) commercial Mouse Inflammation Kit as described in the manufacturer’s protocol. The analysis was performed on a flow cytometer FACS Canto II (BD).

### 2.7. Determination of Intracellular Nitric Oxide in Peritoneal Macrophages

Peritoneal exudate cells were obtained from the ascites liquid. A 50-μL aliquot was taken from the sample and centrifuged at 3000× *g* for 5 min, then the supernatant was removed and 10 µL of the working solution of the 4-amino-5-methylamino-2’, 7’-difluorofluorescein diacetate (DAF-FM DA) (Thermo Scientific, Eugene, OR, USA) fluorescent probe was deposited at a concentration of 5 µM, and incubated at 37 °C for 30 min in the dark. Then, 500 μL of sterile PBS were added and centrifuged at 3000× *g* for 5 min, the supernatant was discarded, 500 µL of PBS were added, and it was incubated at 37 °C for 15 min in the dark. It was then centrifuged at 3000× *g* for 5 min, and the supernatant was discarded [18]. The cell packet labeled with the DAF-FM DA probe was prepared for peritoneal macrophage labeling. In total, 4 µL of the anti-F4/80 monoclonal antibody labeled with APC (Biolegend, San Diego, CA, USA) was deposited and incubated for 30 min at ambient temperature in darkness. Subsequently, 500 μL of FACSFlow was added, centrifuged at 3000× *g* for 5 min, and the supernatant was removed. The cell packet was resuspended in 200 μL of FACSFlow to be evaluated in the FACS Canto II (BD) flow cytometer.

### 2.8. Determination of Lymphocytes Populations in Mice Spleen

The spleen was divided between 2 slides with a frosted edge, with the homogenate being resuspended in 10 mL of PBS, and centrifuged at 500× *g* for 10 min at 4 °C. The supernatant was removed and 5 mL of lysis buffer for red blood cells (NH_4_Cl 0.155 M, NaHCO_3_ 0.01 M, EDTA 0.1 mM) were added, homogenized, and incubated at room temperature for 5 min. Two washes were made with 5 mL of PBS and centrifuged at 500× *g* for 10 min at 4 °C. The supernatant was then discarded and 1 mL of sterile PBS was added. Splenocytes were counted in a Neubauer chamber and adjusted to 1 × 10^6^ cells/mL. Subsequently, the cells were labeled with anti-CD22 monoclonal antibodies conjugated with FITC, anti-CD335 conjugated with PE/Cy7, anti-CD3 conjugated with PE, anti-CD4 conjugated with FITC, anti-CD8 conjugated with APC, anti-CD25 conjugated with PE, and anti-CD44 conjugated with PE/Cy7 (all from BioLegend, San Diego, CA, USA) at room temperature for 30 min in darkness [19]. A FACSFlow wash was performed, and finally, 200 µL of FACSFlow were added. The analysis was carried out on a FACS Canto II flow cytometer (Becton Dickinson). A total of 30,000 events were captured using the BD FACSDIVA software and analyzed with the Flowjo X software [20].

### 2.9. Statistical Analysis

GraphPad Prism^®^ version 6.0 software for Mac was used and the one-way Tukey’s multiple comparisons test was applied. Survival data were presented in a Kaplan–Meier survival curve. A 95% confidence interval was considered significant (*p* < 0.05).

## 3. Results

### 3.1. Clinical Evaluation of Organs

**Figure 1:**
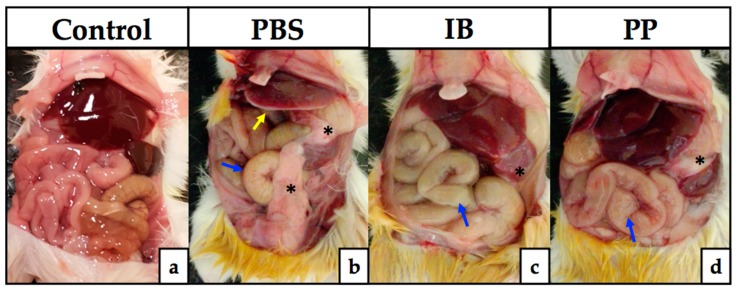
Clinical evaluation of the pathology in peritoneum organs. The development of inflammation of the intestine (blue arrow), appearance of necrotic foci in the liver (yellow arrow), and fibrosis formation around the spleen (black asterisks) are signs of parasite infection on the seventh day post-infection, being more evident in the group treated with PBS (**b**) with respect to those treated with viable *Lactobacillus casei* (**c**) and non-viable Lc (**d**), when compared to healthy mice (**a**). PBS) phosphate buffered saline; IB) immunobiotic; PP) paraprobiotic.

To demonstrate the degree of damage generated by intraperitoneal inoculation of tachyzoites of *T. gondii* RH, the peritoneum organs were exposed. The morphology and typical appearance of the peritoneal organs of healthy CD1 mice showed a total absence of a pathological process (Figure 1a). All groups that were infected with *T. gondii* showed different degrees of organ lesions, with abnormalities in the PBS-treated group being more evident (Figure 1b), with the appearance of necrotic foci in the liver (yellow arrow), bowel inflammation (blue arrow), and fibrosis around the spleen that extended to partially cover the intestines (black asterisks). The groups treated with immunobiotics (Figure 1c) and paraprobiotics (Figure 1d) showed inflammation of the intestines (red arrows) and poor fibrosis, which covered the spleen only (blue asterisks), with a loss of normal liver coloration.

### 3.2. Evaluation of the Parasitic Load in Peritoneal Exudate

**Figure 2:**
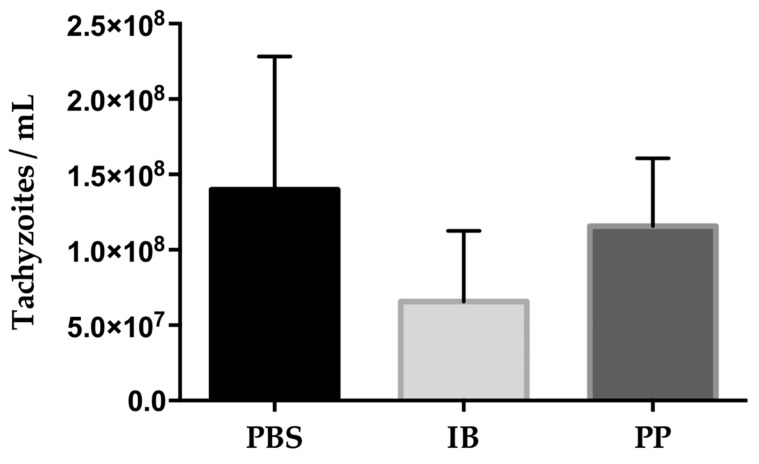
Quantification of *T. gondii* RH tachyzoites in peritoneal fluid. The parasitic load was reduced by 53% in the group treated with immunobiotic and 17.3% in those treated with paraprobiotic compared to the control group treated with PBS. Data were combined from two individual experiments with three to five mice per group. Values represent the means ± SD. No significant difference between treatments. PBS) phosphate buffered saline; IB) immunobiotic; PP) paraprobiotic.

The quantification of tachyzoites in peritoneal exudate was evaluated at 7 days post-infection in infected mice. The control group that received the PBS treatment showed the highest amount of tachyzoites (1.39816667 × 10^8^) while the groups that received the *Lactobacillus* treatment reduced the parasitic load of tachyzoites, 6.5725 × 10^7^ with immunobiotic and 1.15628571 × 10^8^ with paraprobiotic. The reduction of the parasitic face in the immunobiotic group was 53% while the group treated with paraprobiotic was 17.3% with respect to the control group (Figure 2). The quantification of peritoneal exudate cells (PECs) was performed in the peritoneal exudate of infected mice and the relationship with respect to the amount of tachyzoites present was investigated, showing a greater amount of tachyzoites per PEC in the control group (1:47.47). In the groups treated with immunobiotic, the ratio was 1:9.12 and those treated with paraprobiotic was 1:17.5.

### 3.3. Percentage of Activated Macrophages and Production of Intracellular Nitric Oxide

**Figure 3:**
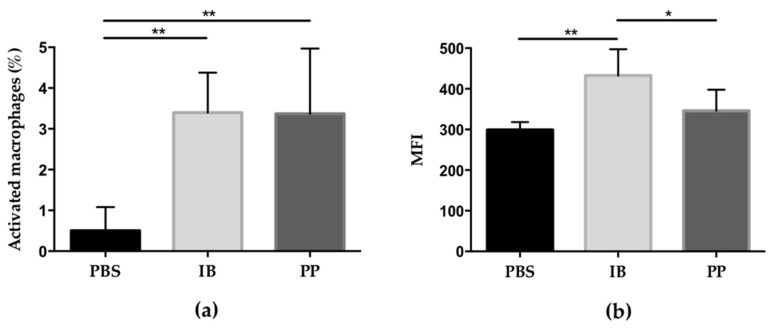
Evaluation of the activation of peritoneal macrophages and production of intracellular nitric oxide in CD1 mice infected with *T. gondii* RH: (**a**) the percentage of activated macrophages in the peritoneal cavity is increased in treatments with lactobacilli, regardless of whether they are alive or dead, compared to the control group; (**b**) there is an increase in the production of nitric oxide in the group treated with immunobiotic compared to the other two groups. Data were combined from two individual experiments with three to five mice per group. Values represent the means ± SD. * *p* < 0.05; ** *p* < 0.01. PBS) phosphate buffered saline; IB) immunobiotic; PP) paraprobiotic; MFI) mean fluorescence intensity.

The percentage of activated macrophages in the peritoneal cavity of mice infected with *T. gondii* was determined. Those cells that expressed double labeling with the anti-F4/80 monoclonal antibody and the DAF-FM DA probe were selected. It was observed that the number of activated macrophages of the control group was less than 1% while in the groups treated with lactobacilli, the percentage was greater than 3%, with there being a significant difference with respect to the control group (Figure 3a). The amount of nitric oxide produced intracellularly was evaluated by measuring the average fluorescence intensity (MFI) generated by the DAF-FM DA probe, which is directly proportional. The macrophages that produced the greatest amount of intracellular nitric oxide were from the mice treated with the immunobiotic with respect to the mice treated with paraprobiotic and PBS (significant difference) while the group treated with paraprobiotic generated higher NO production compared to the control group, although this was not significant (Figure 3b).

### 3.4. Quantification of Cytokines in Ascites Fluid 

**Figure 4:**
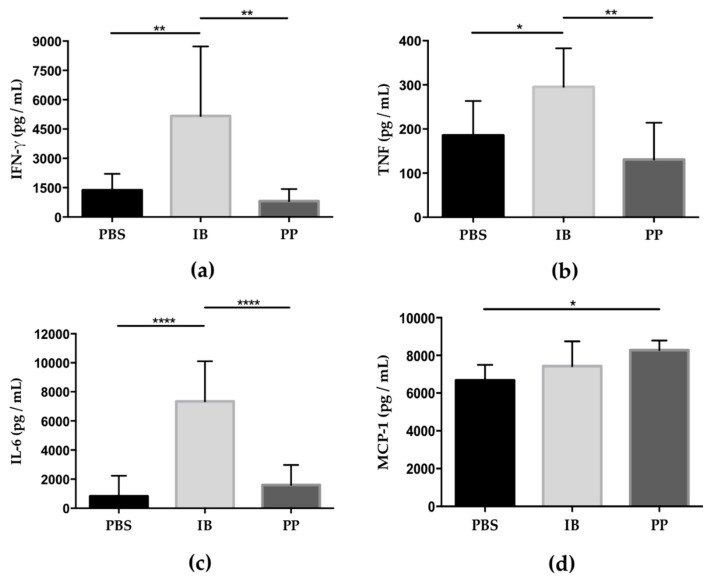
Cytokine levels in the ascites fluid of mice infected with *T. gondii*. Concentrations levels of interferon-gamma (FN-γ), tumor necrosis factor (TNF), and interleukin-6 (IL-6) were significantly elevated in mice treated with immunobiotic (IB) compared to mice in the control group (PBS) and paraprobiotic (PP) but without significant differences between the PBS and PP groups (**a–c**). The administration of the paraprobiotic treatment significantly increased the concentration of monocyte chemoattractant protein-1 (MCP-1) compared to the group treated with PBS, however, no significant difference between the PP and IB groups or between the IB and PBS groups (**d**). Data were combined from two individual experiments with three to five mice per group. Values represent the means ± SD. * *p* < 0.05; ** *p* < 0.01; **** *p* < 0.0001. PBS) phosphate buffered saline; IB) immunobiotic; PP) paraprobiotic.

Proinflammatory cytokine levels in the peritoneal fluid were determined at 7 days post-infection. The levels of IFN-γ, TNF, and IL-6 were significantly increased in mice treated with immunobiotics compared to the groups treated with paraprobiotic and PBS (Figure 4a–c). However, chemokine MCP-1 was observed as significantly increased only in the group treated with paraprobiotic compared to the control group (Figure 4d). The cytokines IL-10 and IL-12 showed no significant differences between the groups.

### 3.5. Determination of Spleen Cell Populations

**Figure 5:**
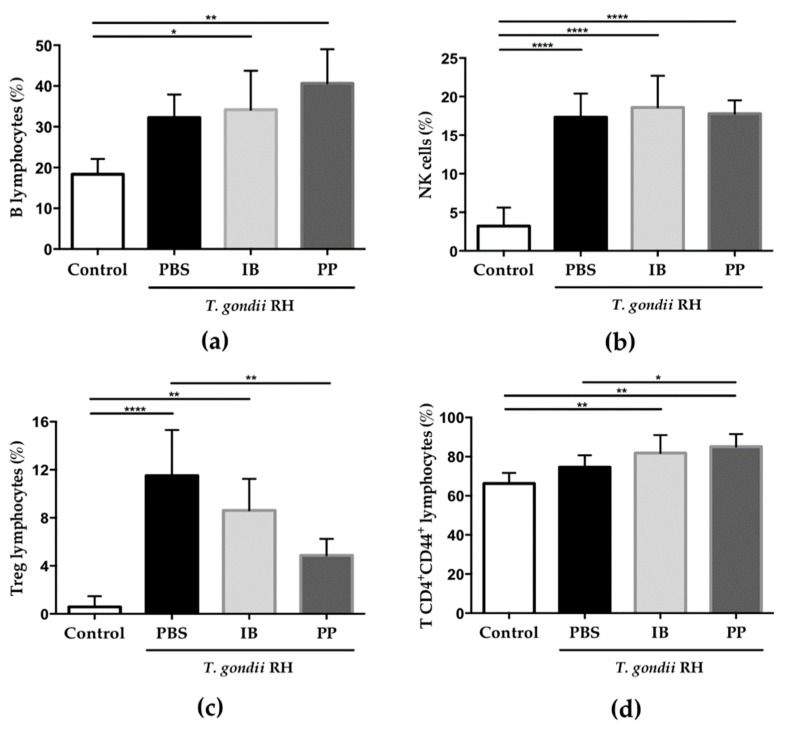
Determination of the percentage of the spleen cell populations on the seventh day post-infection with *T. gondii* RH. Splenocytes were labeled with different monoclonal antibodies for B lymphocytes (**a**), Natural Killer cells (**b**), regulatory T lymphocytes (**c**), and T CD4^+^CD44^+^ lymphocytes (**d**) Data were combined from two individual experiments with three to five mice per group. Values represent the means ± SD. * *p* < 0.05; ** *p* < 0.01; **** *p* < 0.0001. PBS) phosphate buffered saline; IB) immunobiotic; PP) paraprobiotic. PBS) phosphate buffered saline; IB) immunobiotic; PP) paraprobiotic.

Since the immune response during infection with *T. gondii* involves cell populations that must be mobilized from secondary lymphoid organs, some of the most important lymphocyte populations in the spleen were evaluated. The presence of the protozoan increased the percentage of B lymphocytes (Figure 5a) and Natural Killer cells (Figure 5b) while regulatory T lymphocytes (Figure 5c) and the subpopulation of T CD4^+^CD44^+^ lymphocytes (Figure 5d) also showed a significant difference between the treated groups.

### 3.6. Survival of CD1 Mice to Infection with T. gondii

**Figure 6:**
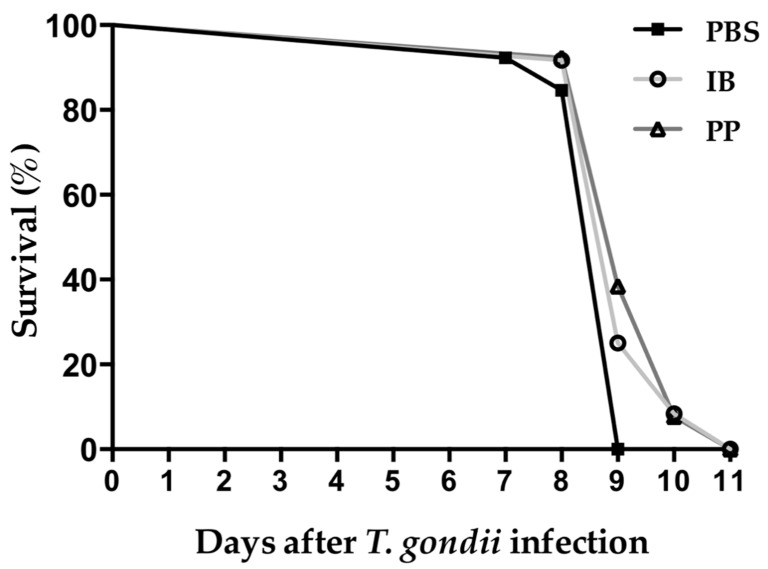
Survival of CD1 mice after infection with *T. gondii*. The mice were infected with 1000 tachyzoites of the RH strain of *T. gondii* intraperitoneally. All of the mice that were treated with PBS died on day 9 post-infection while all mice treated with immunobiotic and paraprobiotic died on day 11 post-infection. Data were combined from two individual experiments with six to seven animals per group. PBS) phosphate buffered saline; IB) immunobiotic; PP) paraprobiotic.

We evaluated the protective capacity of viable and non-viable Lc during a complete course of infection with tachyzoites of *T. gondii* RH. The mice that received the vehicle (PBS) showed signs of piloerection, overcrowding, and hyperventilation from 4 days post-infection, with all animals treated with PBS succumbing at 9 days post-infection. The mice that were treated with lactobacilli had the same signs until day 6 post-infection. At 9 days, there was 25% and 38.5% survival in mice treated with viable Lc and non-viable Lc, respectively. In the end, mice treated with lactobacilli succumbed to infection at day 11 post-infection (Figure 6).

## 4. Discussion

The work shows anatomical, parasitological, and immunological evidence of the ability of live (immunobiotic) and dead (paraprobiotic) lactobacilli to stimulate the systemic immune system to control replication and pathology caused by infection with the protozoan *T. gondii*. The complexity and speed of replication of intracellular protozoa have made it difficult for patients to have effective and safe treatments to date. However, probiotics arise as a possibility that should be evaluated in in vivo models and demonstrate evidence of their benefit to health.

The systemic toxoplasmosis model caused by *T. gondii* RH allowed us to have a possible overview of the immunological mechanisms that a commercial probiotic, such as *Lactobacillus casei*, can carry out regardless of its viability.

The effect of probiotic lactobacilli goes beyond the gastrointestinal tract. It has been shown that the induced immune response in the small intestine can be extended to the systemic immune system and reach distant sites through lymphatic and blood circulation, which connects the intestinal immune system with the systemic immune system [21,22].

The administration of viable and non-viable Lc increased the microbicidal activity of peritoneal macrophages on the tachyzoites of *T. gondii* RH, as well as the cytokine production capacity.

This effect is closely related to the parasitic load, where the mice treated with immunobiotics presented the least amount of free tachyzoites in relation to the control group. High concentrations of IFN-γ and TNF stimulate the activation of peritoneal macrophages as well as the production of intracellular nitric oxide in mice treated with immunobiotics. There are in vitro and in vivo reports that some species of lactobacilli, including *Lactobacillus casei*, stimulate the production of proinflammatory cytokines IFN-γ and TNF [23]. IFN-γ, like TNF, play an important role in the resistance to infection of *T. gondii* due to the ability of macrophage activation [24]. IFN-γ is produced primarily by NK cells, T CD4^+^ cells, and T CD8^+^ cells in response to the antigenic recognition of glycosylphosphatidylinositol by Toll-like receptor 2 and 4 (TLR2 and TLR4), as well as to the profilin-like protein that is recognized by TLR11 [25]. IFN-γ has a pleiotropic effect on infected cells, mainly macrophages that result in the reduction of protozoan replication by inducing the expression of 2,3 dioxygenase indolamine. It depletes tryptophan, which is an essential amino acid for growth of *T. gondii*, increases the expression of inducible nitric oxide synthase (iNOS) through the production of NO, and depletes arginine, which is another essential amino acid for the parasite, and finally, the expression of immunity-related GTPases (IRGs) and guanylate-binding proteins (GBPs) that are responsible for the destruction of the parasitophorous vacuole [26].

Macrophages play a key role in controlling the replication of tachyzoites of *T. gondii*. One of the main microbicidal mechanisms of activated macrophages is the production of nitric oxide. However, it is known that *T. gondii* has the ability to evade the cytotoxic effect of nitric oxide by inhibiting the production of NO in mouse activated macrophages [27,28]. Cabral [29] suggest that inhibition of NO production in activated macrophages infected with *T. gondii* is a general phenomenon, but the suppression of iNOS varies depending on the macrophage cell line. The increase in the percentage of activated macrophages, as well as the production of intracellular nitric oxide in mice treated with immunobiotics, show that inhibition of NO production by *T. gondii* can be reversed after administration of viable Lc, thereby increasing the microbicidal capacity of activated macrophages.

Interestingly, spleen cell populations increased when mice were infected with *T. gondii*, however, regulatory T lymphocytes were found to be decreased and T CD4^+^CD44^+^ cells increased in the groups treated with Lc relative to the control group. IL-10 is the main cytokine that regulates the inflammatory process generated by *T. gondii*, so increasing the population of this cell is indicative of the severe immunopathology carried out in the peritoneal organs of the control group [30]. For T CD4^+^CD44^+^ lymphocytes, it has been shown that they have the ability to mobilize towards the flash point [31,32].

The prolongation of the survival of the mice treated with Lc showed that there is a greater control and elimination of tachyzoites of *T. gondii*, but the inflammatory process is so severe that the immunopathology caused led to the death of the mice treated with Lc, and this is due to the high replication of the parasite, where the *T. gondii* strain RH had a doubling time of approximately 5 h [33].

## 5. Conclusions

This is the first study carried out that demonstrates the remote immunostimulation of a viable and non-viable probiotic on a murine systemic toxoplasmosis model. The results demonstrate that regardless of the viability of *Lactobacillus casei*, these can activate macrophages and destroy tachyzoites by producing intracellular nitric oxide. This study lays the groundwork to investigate the molecular mechanism of immunostimulation of paraprobiotics on lethal systemic parasitic infections.
